# Climate-Driven Adaptation, Household Capital, and Nutritional Outcomes among Farmers in Eswatini

**DOI:** 10.3390/ijerph16214063

**Published:** 2019-10-23

**Authors:** Karen M. Bailey, Robert A. McCleery, Grenville Barnes, Sarah L. McKune

**Affiliations:** 1 University of Colorado Boulder Environmental Studies Program, University of Colorado Boulder, 4001 Discovery Dr., Boulder, CO 80303, USA; 2 Department of Wildlife Ecology and Conservation, University of Florida, 110 Newins-Ziegler Hall, Gainesville, FL 32611, USA; ramccleery@ufl.edu; 3 School of Forest Resources and Conservation, University of Florida, 136 Newins-Ziegler Hall, Gainesville, FL 32611, USA; gbarnes@ufl.edu; 4 Department of Environmental and Global Health, University of Florida, 1225 Center Drive Gainesville, FL 32610, USA; smckune@ufl.edu

**Keywords:** climate change, adaptation, drought, capital, nutrition, Eswatini, anthropometrics

## Abstract

Globally, communities are increasingly impacted by the stressors of climate change. In response, people may adapt to maintain their livelihoods and overall health and nutrition. However, the relationship between climate adaptation and human nutrition is poorly understood and results of adaptation are often unclear. We investigated the relationship between adaptation and child nutrition, in Eswatini (formerly Swaziland) during an extreme drought. Households varied in both adaptation behavior and household resources and we found that, overall, households that adapted had better child nutrition than those that didn’t adapt. When controlling for the influence of household capital, we found that more vulnerable households, those with greater dependence on natural resources and lower income, had a stronger positive relationship between adaptation and nutrition than less vulnerable households. We also found that some adaptations had stronger positive relationships with nutrition than others. In our system, the adaptation that most strongly correlated with improved nutrition, selling chickens, most likely benefits from local social networksand consistent demand, and performed better than other adaptations. Our results emphasize the need to measure adaptation outcomes and identify and support the types of adaptations are most likely to improve nutrition in the future.

## 1. Introduction

Climate change is negatively impacting communities across the globe, with implications for livelihoods and human health and nutrition [1,2,3]. In response to changing conditions, households adjust their livelihoods - the resources and daily activities necessary to make a living [1,4,5,6]. Ideally, these adjustments and adaptations mitigate the negative impact of climate change and allow people to maintain the overall health and nutrition of their household [7,8,9].

There are few empirical studies examining the direct relationship between adaptation and nutrition [7,9,10,11]. Previous research has focused on understanding the relationship between household nutrition and household resources, or relationships between household adaptions and its capital [12,13]. Household capital is important for adaptation and nutrition, as households with greater human capital (for example, education, employment), financial capital (for example, wealth, assets), and social capital (for example, connectedness within social networks) often report better health [14,15,16]. And while household capital is critical for adaptation and nutrition, researchers have rarely evaluated adaptation as a direct mechanism for influencing nutrition. We would expect climate adaptation to influence nutrition in a number of ways. Adaptation is likely to influence farming practices, livelihood strategies, income, daily activities, and potentially food security [10,17,18,19]. Through these livelihood adaptation the number of meals, amount of food, types of food, and diversity of food consumed could all be altered, influencing nutritional outcomes. In cases where linkages are made between livelihood adaptations and nutrition, there is often a narrow focus on agricultural practices and nutritional outcomes, rather than considering a broad suite of potential adaptation options [20,21]. To fully understand the relationships between climate adaptation and nutrition, we must explicitly examine how households adapt their livelihoods in respond to changing climatic conditions, not just their farming practices. Understanding this relationship is necessary to identify and support adaptations that are most likely to help people survive and thrive in the future [7,22,23].

Understanding the relationships between adaptation and nutrition is especially critical for rural farming communities in low- and middle-income countries. These communities are heavily dependent on natural resources, which makes them especially vulnerable to impacts of climate change [24,25]. While a number of climate-related threats impact these communities, including flood, increased variability in seasonality, increased frequency and intensity of extreme events, and rising CO_2_ levels, drought, in particular, poses a major threat to resource dependent livelihoods across the world [26,27,28]. Further the frequency and intensity of drought is expected to worsen in the coming decades with significant implications for livelihood sustainability in arid and semi-arid environments [29,30,31]. For resource-dependent communities in low- and middle-income countries, successful drought adaptation is essential for their long-term nutrition and survival [32,33,34].

Nutrition is critical to understand, as it lays the foundation for other life pursuits [35,36,37]. People who are undernourished are often unable to work, socialize, procreate, or pursue other personal, emotional, or mental goals [38,39,40,41]. Good nutrition is achieved when sufficient nutrient-rich foods are consumed, a healthy living environment is secured, and the absence of disease (clinical or subclinical) supports optimal growth and health. Malnutrition–specifically, undernutrition–occurs when bodily functions such as growth or disease recovery fail to reach their full potential due to physical impairment [42]. Malnutrition has been a problem in developing countries for decades and is a leading cause of child and infant mortality, particularly in Sub-Saharan Africa [43,44,45]. Malnutrition can also negatively impact long-term growth, cognition, lifetime achievements, and health of future generations [36,46]. In particular, children under five are at greater risk of malnutrition than older demographics, and their nutritional status responds strongly to acute changes such as drought, famine, and conflict [47]. Child nutrition is associated with household capital, specifically human and financial capital [48,49,50]. Households with less capital, and less diverse capital tend to be more vulnerable to climate stress [13,51]. Understanding household capital allows us to identify the most vulnerable populations, those likely to suffer under changing climatic conditions and with the greatest need to adapt. To fully understand the impacts of adaptation on child nutrition, it is critical to account for the influences of household capital and vulnerability.

To understand the relationships between adaptation, capital, and nutrition, we studied drought and livelihoods in Eswatini (formerly Swaziland). The specific objectives of this study are (1) to examine whether livelihood adaptations to drought are positively associated with improved nutrition in children and (2) to determine if certain adaptations have stronger positive associations than others with improved nutritional outcomes of children. We predicted that there will be a positive relationship between adaptation and nutrition status.

## 2. Materials and Methods

### 2.1. Study Design

We conducted our research from 2015 to 2016, when Eswatini experienced one of the most severe droughts in recent memory [52]. While Eswatini is expected to experience other negative impacts of climate change, including rising temperatures and increased flood events, drought is of particular concern as it severely impacts food availability and livelihoods and is likely to become more frequent under climate change [53]. Additionally, our initial data collection in the region community members cited drought as the most severe climate challenge facing communities. As such we focus on drought adaptation. After collecting initial information through focus groups, we developed a household survey to collect information on household demographics, measures of the each of the five capitals, adaptation, and nutrition (Appendix A). We translated the survey instrument from English to SiSwati, then back translated it into English to ensure that it was understandable and locally appropriate. All household surveys were collected in SiSwati by a field assistant, fluent in English and SiSwati.

### 2.2. Study Area

Eswatini is a small subtropical country with a population of about 1.4 million and an area of 17,565 km^2^ [54]. It has a mean annual rainfall between 500 and 1500 mm, and ranges in average temperature from 16–22 °C [54]. Historically, Eswatini has a wet and dry season and is characterized by thunderstorms in the summer and decreased precipitation in the winter. 70–80% of the population is either directly engaged in or associated with homestead-based farming [55]. Communal grazing land is traditionally used for cattle. This dependence on crops and cattle is embedded in the social, cultural, and economic landscape of Eswatini and perceived as critical to the country’s success and development [56].

**Figure 1:**
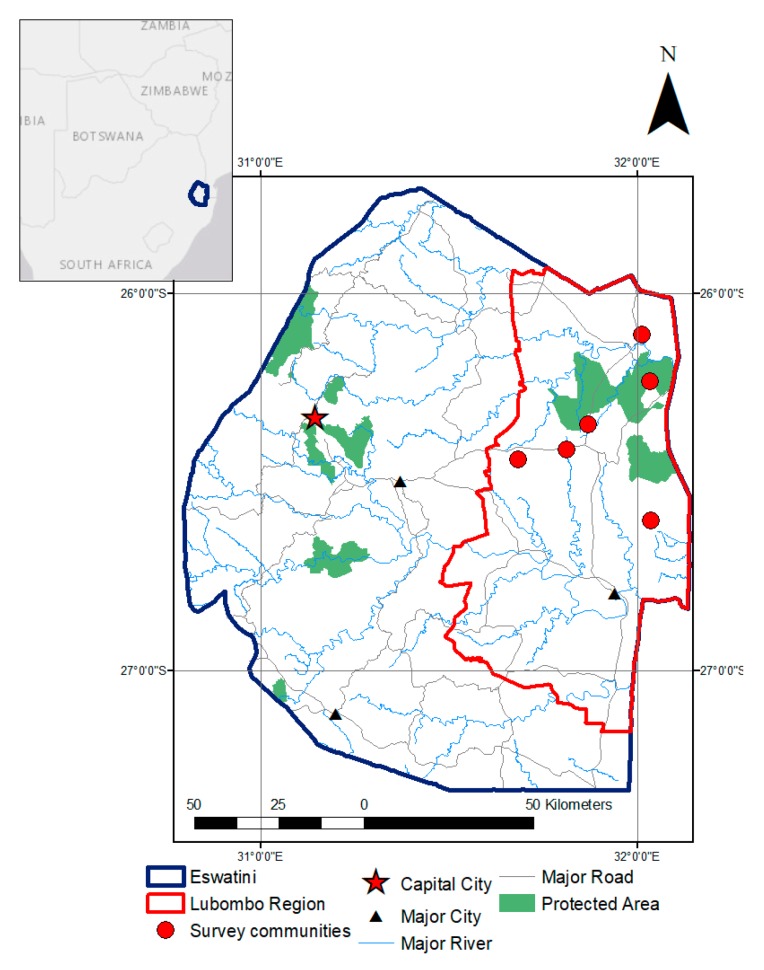
Study area, Eswatini and surrounding countries. The Lubombo region of Eswatini with the capital and major cities marked. Red circles are the study communities. Black lines are major roads, and blue lines are rivers.

We conducted our research in the Lubombo district of eastern Eswatini (Figure 1). This district is comprised of a mosaic of intensive agriculture, protected areas, subsistence cropland, grazing land, and human settlements [57]. Sugarcane plantations are the main commercial agriculture, while maize is the primary subsistence crop [58]. The research team worked in 6 communities, 3 in the Lowveld (Mpaka, Matsetsa, and Lonhlupekho) and 3 in the Lubombo Mountains (Shewula, Mhlumeni, and Luketseni, Figure 1). These communities were selected after consultation with local residents to reflect the variation in infrastructure, agro-ecology, and population size of the region. Within each of the communities, we randomly selected 50 homesteads to include in the study, for a total of 300 household surveys.

### 2.3. Preliminary Data

During our focus groups, eight primary adaptation strategies typically employed in response to drought were identified: planting drought/heat resistant crops, using conservation farming methods to minimize soil erosion, keeping bees to sell honey, chicken husbandry, selling natural resources, selling handicrafts, pursuing off-farm work, and participating in training and capacity building provided by aid organizations. In our survey, each respondent indicated whether their household had ever performed any of the listed adaptation strategies, if they had performed it within the past 5 years, and what resources were necessary to perform the adaptation (time, money, land, labor, etc.). To determine if an adaptation was successful, the respondent indicated, in his/her opinion, whether the adaptation led to a change in household food consumption, income, or agricultural output. We categorized an adaptation that led to an increase in any of the three desired outcomes as successful. For each household, the following indicators were tallied: the total number of adaptations attempted, the ratio of successful adaptations to attempted adaptations, and a binary measure of whether or not they had any successful adaptations [59].

To assess human capital during household surveys, we asked questions about household demographics (e.g., age, highest level of education, employment status). For natural capital, we asked households to report access to and use of natural resources (use of wild foods plants/animals, distance and method travelled to collect wild foods and firewood), farming activities, yields, and livestock ownership. For financial capital we asked households to report income from all sources (employment, small businesses, and craft/natural resource sales), remittances, and expenditures related to health, groceries, and school fees. For physical capital we asked households to list ownership of locally relevant physical assets (including vehicles, farming tools, and electronics), and access to and use of other physical resources and infrastructure (e.g., electricity, type of water source, distance to hospitals and schools). Last, for social capital, we collected data on participation in community groups, attendance at community meetings, hiring or working for neighbors, trade or exchange with neighbors, and household positions of leadership (Appendix A).

Our initial research revealed that social and natural capital were strongly associated with adaptation success and measures of physical and financial capital were also positively associated [59]. Specifically, we found positive associations with adaptation with the following characteristics (by capital): a household’s participation in a community organization and working for or hiring neighbors (social capital); total land available for farming, distance to savanna resources, and owning livestock (natural capital); access to running tap water and electricity (physical capital); and income (financial capital). To eliminate confounding effects, we controlled for these variables in models with nutrition as a response variable.

### 2.4. Nutrition

Recognizing that it is an imperfect indicator, we used nutritional status of children under five as a proxy for the household’s overall nutrition [60,61,62]. To measure nutritional status, we used anthropometric measures, including children’s weight and height. Ultimately, weight-for-age, weight-for-height, and height-for-age z-scores were calculated and compared against international reference scores. We used a weight-for-height z-score (WHZ), where two standard deviations below the international reference, or wasting, was an indicator of acute malnutrition which might be caused by drought or other acute, severe disturbance in food supply [60,63]. We also used a height-for-age z-score (HAZ), where two standard deviations below the international reference, or stunting, was also an indicator of chronic malnutrition. Stunting can be caused by a range of chronic drivers, including limited caloric intake, poor quality diet, poor hygiene and sanitation, and infection and accrues over a longer period of time [64,65,66,67,68]. We also used a weight-for-age z-score, where two standard deviations below the global reference was considered underweight. This metric is used to indicate a chronic condition that contributes to increased illness and mortality, but because it is calculated based on weight it is the most temporally fluid of the three metrics (age, weight, and height) and can reflect acute food shortages or illness [44,50]. Because WAZ is the most fluid and responsive metric of the three, we predict that it will have stronger significant relationships with adaptation behavior than HAZ or WHZ.

At the end of each survey, we recruited all children under five years old within each household for inclusion in the nutritional component of the research. We recorded sex, birthdate, weight, and height for each child using established methods [69,70]. We generated Z-scores for each of the three indicators (WHZ, WAZ, and HAZ) for each child measured in the household [71,72]. We also collected data on meals missed and food consumed during the past 24 h and recent consumption of animal sourced foods.

### 2.5. Statistical Analysis

We conducted our analyses using RStudio and the R platform [73,74]. We used generalized linear models to test the relationships between adaptation, capital, and nutrition. Using the R packages Igrowup and Anthro, we calculated a WHZ, WAZ, and HAZ for each child in the household [72,75]. We calculated Z-scores according to the World Health Organization as:

$$\frac{the\;observed\;value-median\;value\;of\;the\;reference\;population}{standard\;deviation\;value\;of\;the\;reference\;population}$$

For households with more than one child under five (27% of households), z-scores of the youngest child was used to generate a single nutritional indicator for the household [76]. This resulted in some data loss for some households but still yielded useful information as youngest children are often more vulnerable to negative outcomes associated with malnutrition [77].

#### 2.5.1. Adaptation and Nutrition

We generated four generalized linear regression models for each measure of household nutrition (HAZ, WAZ, and WHZ) as the response variable. Because z-scores were normalized by subtracting the median value and dividing by standard deviation, they have a Gaussian distribution. For all models, we treated all predictor variables as fixed effects. The first model included total attempted adaptations as a predictor variable. The second model included the ratio of successful to attempted adaptations as a predictor variable. The third included whether a household had a successful adaptation as a predictor variable. We compared the goodness of fit of each of the four models based on R-squared values. Within each model, we evaluated the strength of each predictor variable based on its standardized beta coefficient and *p*-value of the Wald test [78].

#### 2.5.2. Adaptation, Nutrition, and Capital

We controlled for the influence of capital through the use of random effects in a generalized linear mixed effects model. We had previously identified social, natural, physical, and financial capital as statistically significant predictors of adaptation [59]. For each type of capital, we identified which indicator variable(s) had the strongest relationship with adaptation based on beta coefficients, *p*-values, and principal component loadings [59]. We then included these variables as random effects in models with nutrition as an outcome variable and adaptation behavior as a predictor. We compared the goodness of fit of each model based on R-squared values. Within each model, we evaluated the strength of each predictor variable for the two datasets, based on its standardized beta coefficient and *p*-value of the Wald test [78].

#### 2.5.3. The Role of Individual Adaptations

Finally, we ran generalized linear mixed regression models for each measure of malnutrition (HAZ, WAZ, and WHZ) as the response variable. The models included a fixed binary variable indicating whether a household attempted each of the eight adaptations we surveyed. In each of these regressions, we included variables likely to either impact nutrition or influence the relationship between adaptation and nutrition as random effects. For example, households that sell chickens may also have increased access to protein, which could mask relationships between selling chickens as an adaptation and consuming chicken. Similarly, households that collect and sell natural resources such as firewood, may be less likely to have access to electricity and lower overall incomes which may confound the relationship between the adaptation and nutrition. Including these variables as random effects allows clearer interpretation of the relationships between adaptation and nutrition. The influence of the success of all eight adaptations on nutrition could not be tested, due to small sample size; too few households had both successful individual adaptations and children under 5 measured. We assessed these relationships when there was a minimum of 30 households that reported adaptation success and also had children under 5. We assessed the goodness of fit of each model based on R-squared values and standardized beta coefficient and *p*-value of the Wald test (Engle, 1983).

Prior to data collection, this research was approved in accordance with Institutional Review Board protocols at the University of Florida (UFIRB 2015-U-1348).

## 3. Results

**Figure 2:**
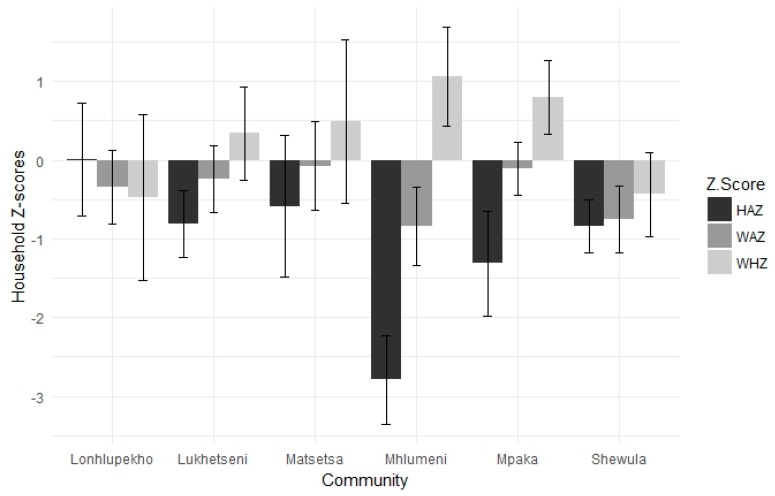
Average z-scores for children under the age of five in households across the six survey communities in the Lowveld and Lubombo regions of Eswatini.

The average WAZ was −0.41 (SD = 1.8); the average HAZ was −1.16 (SD = 2.1), and the average WHZ was 0.34 (SD = 2.3). Extreme malnutrition of any type (indicated by a z-score below 3) occurred in 24% of children, with extreme stunting being the most common (15%, Table 1). Undernutrition and wasting were comparable across the six communities, with a total average of 16% and 15%, respectively (Table 1). Among the six communities, Shewula had the lowest average z-scores for both (Figure 2). Thirty-two percent of children in our survey population were stunted. Average HAZ by community were negative in all communities except Lonhlupekho and were lowest in Mhlumeni (Figure 2).

### 3.1. Adaptation and Nutrition

Households attempted planting heat and drought resistant crops most often (Table 2). The adaptation with the greatest reported success rate was collecting natural resources (primarily firewood) to sell and bee keeping had the lowest success rate. The most common type of success was increased income; 65% of households that reported success in any adaptation reported increased household income. The least common type of success was increased food consumption; only 9% of households that reported success in any adaptation reported increased household food consumption. Increased agricultural output was reported by 26% of households reporting success. Adaptations varied significantly in their required inputs, potential outputs, and daily activities (Appendix B).

Overall, nutritional status of children was positively correlated with attempted adaptations, negatively correlated with the ratio of successful to attempted adaptations, and not correlated with the probability of having a successful adaptation (Table 3). As predicted, across measures of adaptation, WHZ and WAZ were significantly associated with adaptation, while HAZ, the nutritional indicator reflecting chronic rather than acute responses, did not (Table 3). WHZ was marginally positively correlated with attempted adaptations (β = 0.34, *p* = 0.06) and negatively correlated with the ratio of successful adaptation to attempted adaptations (β = −1.77, *p* = 0.03). WAZ was negatively correlated with the ratio of successful adaptation to attempted adaptations (β = −1.22, *p* = 0.052).

### 3.2. Adaptation, Nutrition, and Capital

For households with less wealth (those that spent less than $30 per month on groceries), adaptation attempts, an indicator reflecting the number of adaptations the household reportedly attempted, was marginally positively associated with WHZ (β = 0.53, *p* = 0.055). This trend did not hold for wealthier households, which had no relationship between adaptation attempts and nutrition Among households with more farmland, a stronger relationship was found between adaptation attempts and WAZ (β = 0.31, *p* = 0.041) than those with less farmland (β = −0.18, *p* = 0.46). When controlling for the influence of other capital metrics, there were no significant differences in relationships between adaptation and nutrition.

### 3.3. The Role of Individual Adaptations

**Figure 3:**
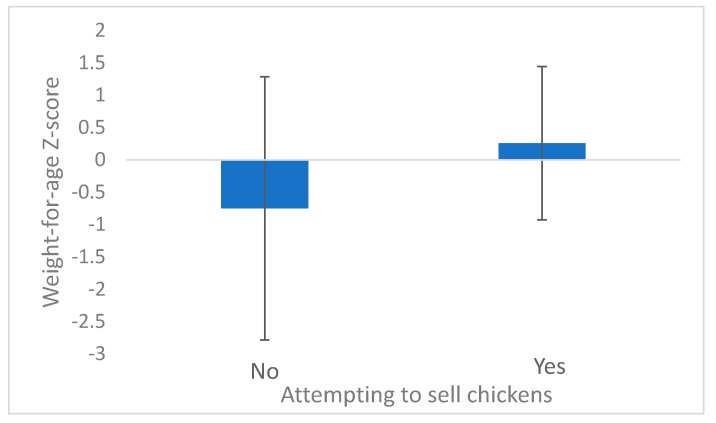
Weight-for-age z-scores for households that attempted to sell chickens and households that did not (*p* = 0.004).

**Figure 4:**
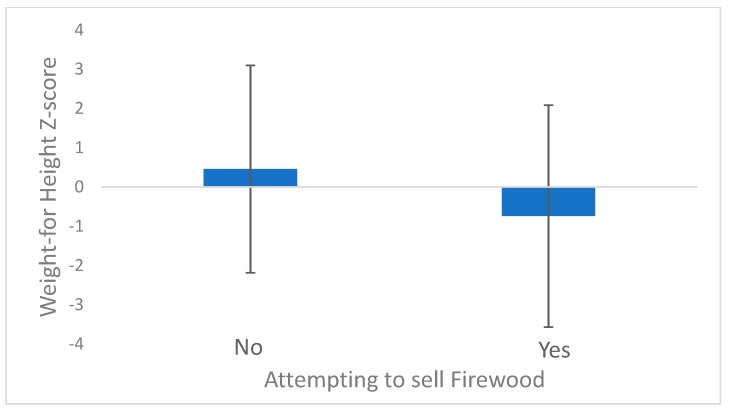
Weight for height z-scores for households that attempted to sell firewood and households that did not (*p* = 0.045).

Selling chickens as an adaptive strategy was positively correlated with WHZ (β = 1.35, *p* = 0.013) and WAZ (β = 1.11, *p* = 0.003, Figure 3). Conversely, attempting to sell firewood was negatively correlated with WHZ (β = −1.23, *p* = 0.045, Figure 4). Other than those two adaptations, no others had statistically significantly impacts on nutrition based on success. Our sample size limited our ability to detect these relationships.

### 3.4. Drivers of Adaptation

While most households (70%) reported improved livelihood conditions as a result of adaptation behavior, many households reported a number of interlinked multi-scalar drivers associated with the decision to adapt. For many households, the decision to establish a small business or change farming practices was driven by engagement with a community organization, NGO, or government representative. Several of these organizations and outreach efforts were newly formed, in response to the drought or fears associated with the drought. Others were formed to alleviate poverty, promote food security, and support sustainable alternative livelihoods. Other adaptations were initiated individually, leveraging on social capital to take advantage of a new opportunity (such as access to new markets or mastery of a new skills, Appendix B). In many cases, these new ventures (selling crafts or chickens, for example) were supported by financial capital provided by organizations that intervened in anticipation of climatic changes. In such instances, the household wasn’t acting in response to or anticipation of drought but other entities were. The multi-faceted, multi-scalar drivers of adaptation behavior we observed are common across contexts [79,80,81]. While these complex interactions make it challenging to explicitly link drought, behavior change, and nutrition in our study, they do provide useful context for understanding how drought influences behavior across scales.

## 4. Discussion

### 4.1. Adaptation, Nutrition, and Capital

We found that adaptation behavior was positively associated with nutritional status (weight for age, weight for height, and height for age) [49,60,82]. This relationship was stronger for shorter-term nutritional measures of wasting and undernutrition than the longer-term nutritional measure of stunting.

Adaptation attempts were positively correlated with average household nutrition. Households that attempted multiple adaptations were more likely to have better child nutritional status than those that attempted only one adaptation. Attempting multiple adaptations is a means of diversifying livelihood strategies and, likely, increases their chances of successful adaptation. Diversifying livelihoods strategies included starting business, adjusting farming activities, and strengthening social networks by participation in community organizations. And while we didn’t see a direct positive link between adaptation success and nutrition, this may be due to the small number of households that reported multiple successful adaptations. Despite that, the link between adaptation attempts and nutrition suggests that over time, attempting diverse livelihood strategies are more likely to decrease vulnerability to climate change and provide households with greater resilience [83,84,85]. Regardless of success, the act of attempting to adapt could have positive consequences for household health. Adaptation often involves obtaining resources and information, and overcoming psychological barriers [59,86,87]. For example, if a household wants to sell chickens, they first must learn what is required to keep surplus chickens, create a connection with someone else who can provide the initial chickens, and gain access to financial capital to buy, feed, and house chickens. They then have to market the chickens to their community. Even if this process ultimately fails, the steps involved provided opportunities to strengthen social networks, identify new sources of capital, and learn, all of which are associated with improved nutrition and health [16,88,89].

The importance of livelihood diversification and the process of adaptation may also explain the negative relationship we observed between the ratio of successful to attempted adaptations and nutritional outcomes. Even if a household is not successful, if they attempted multiple livelihood adaptations, they likely developed and strengthened access to multiple networks and multiple sources of capital, with potential long-term implications for livelihoods. In contrast, those who attempted only one adaptation only built a single network and likely developed fewer pathways for future adaptation and access to capital. Households that had 100% adaptation success were also more likely to have attempted only a single adaptation. This is the case regardless of a household’s income or dependence on natural resources. These households did not diversify across different types of adaptations and households that attempted fewer adaptations and had higher ratios of successful adaptations to attempted adaptations had lower nutrition levels.

The relationships between nutrition and adaptation were stronger when we controlled for household capital. Households with more farmland and less wealth were more likely to have positive relationships between adaptation attempts and nutrition than households with less farmland and more wealth. Households with more land and a greater dependence on farming as a livelihood strategy are more vulnerable to climate impacts and more likely to experience the negative impacts of drought and, thus, the consequent need to adapt in response [63,90,91]. Our data show that households that are not environmentally dependent should be less vulnerable to drought and their health should have a weaker response to adaptation attempts. Households that did not have a history of farming in the region, but had family members working in full-time, off-farm employment reported being less impacted by drought and believed they were better able to cope with drought than other community members. These households could buffer against nutritional impacts of drought by using cash and assets to purchase food.

### 4.2. The Role of Individual Adaptations

Of all eight of the adaptations investigated in this research (Table 2), attempting to sell chickens as an adaptive strategy was associated with better nutritional status, while selling firewood was associated with lower nutritional status. In Eswatini, most households reported supplemental feeding of their chickens, and most people preferred to house chickens in a coop (personal communication). This means that only households with the money and assets to feed and house chickens can keep enough chickens to maintain a small business selling them (Appendix C). Based on field observations, households mostly sell chickens to their neighbors and the demand for chicken was consistent. In contrast, households that sell firewood often sell to people who live outside the community and households that don’t live close to savanna resources. As a result, sales and demand were more variable. Second, households reported selling their chickens for between 35 and 200 rand ($3.50–$14), depending on the size, sex, breed, and market (Appendix B). In a region where the average monthly income is less than 1000 rand ($70), sales can have significant impacts on household income [92]. In contrast, firewood typically sells for 15 to 50 rand and many households reported a declining market for firewood, saying “people collect their own firewood” or “people can’t pay for firewood”. Finally, households also reported that firewood collection was very labor intensive and, in some cases, impractical because of declines in tree resources. Selling chickens seems to be a more efficient and lucrative adaptation than selling firewood, with greater potential for positive impacts on nutrition.

Many adaptations that households reported being successful in the past were not correlated with nutritional status of children. Specifically, planting drought/heat resistant crops and practicing conservation farming had no relationship with child nutrition. We suspect that this was due to the severity and duration of the 2015–2016 drought in Eswatini [52,93]. According to state-level and organizational estimates, this drought was more severe than most and very few small-scale farmers had meaningful crop yields regardless of the strategies and practices used [53,94]. When traditional livelihood strategies were unsuccessful, livelihood diversification into sectors that did not depend exclusively on natural resources were critical for maintaining nutritional health and food security [88,95,96]. These include raising animals that don’t depend on grazing land (chickens), and seeking out opportunities to participate in local organizations to build skills and leverage shared resources for business enterprises [59]. Climate interventions and future research should consider the importance of non-farm livelihood diversification for maintenance of nutrition. Further, work that investigates the relationship between adaptation and nutrition should explicitly consider non-farm adaptations, especially when farming conditions are sub-optimal.

## 5. Conclusions

The goal of this research was to better understand linkages between adaptation, capital, and nutrition. We intentionally included a wide range of potential adaptation options to broaden this understanding and provide a more robust picture of livelihoods and climate change. We found that, despite variation in household resources, adaptation was positively correlated with nutritional outcomes. Further, households that were more vulnerable to climate-related shocks were most likely to have positive relationships between adaptation and nutrition. We also found that non-natural resource-based adaptation is especially important during times of shock. Our findings are useful in understanding adaptation as a mechanism to improve nutrition. However, our findings were drawn from a cross sectional study and thus no conclusions can be drawn about causation. Longitudinal data that can directly measure the inputs and outputs of adaptation and their influence on nutrition and other metrics of health and wellbeing will provide additional insight into the importance of adaptation. Further, future research should examine how adaptation varies across spatial and temporal scales and focus on isolating the direct effects of adaptation.

This research contributes to our understanding of the significance of adaptation and provides promising support for its capacity to promote household nutrition in a context of climate change. To aid the efforts of NGOs, governments, and other organizations operating to improve climate resilience, we identified adaptations that were most likely to positively impact nutrition and wellbeing, even when crop yields were low. This research underscores the importance of interventions that consider local factors that might influence the success or failure of adaptations (social capital and networks, gender norms, economic markets, etc.) and their impacts on nutrition to yield the most effective and efficient adaptations. Ultimately, responding to global change requires a holistic examination of the drivers and consequences of adaptation and this work provides a nuanced understanding of adaptation as a mechanism for nutrition improvement in context.

## Figures and Tables

**Table 1:** Summary of household z-scores for children under five in households in the Lubombo region of Eswatini and proportion of children with z-scores below −2.

		Average Z Scores (SD)			N Malnourished (%)		
Age (Months)	N	HAZ	WAZ	WHZ	HAZ < −2	WAZ < −2	WHZ < −2
0–12	47	−0.39 (2.7)	0.21 (2.4)	1.08 (2.9)	11 (23)	8 (17)	6 (13)
13–24	51	−1.47 (2.2)	−0.42 (1.9)	0.39 (2.5)	19 (37)	9 (18)	10 (20)
25–36	43	−1.91 (1.7)	−0.31 (1.5)	0.91 (1.8)	22 (51)	6 (14)	2 (5)
37–48	53	−0.60 (2.2)	−0.69 (1.4)	−0.55 (2.3)	10 (19)	8(15)	14 (26)
49–60	55	−1.39 (1.5)	−0.67 (1.5)	0.19 (2.0)	15 (27)	9 (16)	5 (9)
Total	249	−1.16 (2.1)	−0.41 (1.8)	0.34 (2.4)	77 (31)	40 (16)	37 (15)

**Table 2:** Proportion of households (total) attempting each adaptation and proportion of those that attempted andreported success (measured as increase in income, food consumption, or agricultural output).

Adaptation	Households Attempting (N)	Reported Success Rate
Planting drought/heat resistant crops	51% (152)	28% (43)
Conservation farming	30% (88)	33% (29)
Bee keeping	6% (19)	5% (1)
Chicken husbandry	23% (69)	67% (46)
Selling natural resources	19% (55)	98% (54)
Selling crafts	22% (66)	86% (57)
Other salaried off-farm work	24% (72)	32% (23)
Participating in local organizations	17% (50)	36% (18)

**Table 3:** Beta estimates for all significant (*p* < 0.05) relationships between nutrition z-scores and adaptation.

Predictor Variables	Weight for Age (Undernutrition)	Weight for Height (Wasting)	Height for Age (Stunting)
Adaptation Attempts	-	0.34	-
Ratio of successful to attempted adaptations	−1.28	−1.77	-

## Supplementary Materials

Supplementary File 1

## Appendix A. Household Survey

Enumerator Name	Translator name
Date of Survey	Community Name
Time survey start	Time Survey End
Sex of Interviewee(s)	Household head (Y/N)
Household Code	

**Section A-Homestead Data**		
A.1	Date of Settlement at homestead	│_____________________________________│
A.2	What is the main type of roof?	
	1. Thatched Grass	
	2. Corrugated Iron	
	3. Other (specify)	│_____________________________________│
A.3	What is the main type of floor?	
	1. Mud	
	2. Cement/stone	
	3. Other (Specify)	│_____________________________________│
A.4	What is the main type of floor?	
	1. Mud	
	2. Cement/stone	
	3. Other (Specify)	│_____________________________________│
A.5	What is the main type of wall?	
	1. Mud and sticks	
	2. Mud and stones	
	3. Cement, cinderblock	
	4. Other (specify)	│_____________________________________│
A.6	Do you have electricity at the homestead?	
	1. No	
	2. Yes	│_____________________________________│
A.7	What fuel is used to cook with?	
	1. Dung	
	2. Wood	
	3. Coal/charcoal	
	4. LPG	
	5. Kerosene	
	6. Electricity	│_____________________________________│
A.7	How many children <16 years old live at the homestead?	│_____________________________________│
A.8	Answer the Following for All Family Members 16 and Older	

**HH ID**	**ID Code**	**Sex**	**Age**	**Marital Status**	**Literate (Yes/No)**	**Years of Education**	**Months/Years at Homestead**	**Principal Occupation**	**Secondary Occupation**
	01								
	02								
	03								
	04								
	05								
	06								

B.1	What is the primary livelihood activity? 1. Own farm activity 2. Salaried employment 3. Agriculture employment 4. Small business 5. Natural resource collection 6. Craft manufacture 7. Other (Specify)
B.2	What is the secondary livelihood activity? (Use codes from above
B.3	What is the tertiary livelihood activity? (Use codes from above)
B.4	Does the primary livelihood activity account for more than 50 percent of your household’s livelihood? 1. No 2. Yes
B.5	Who is the main breadwinner in the household? (Use individual ID Code)
B.6	For household members who work off-farm, how far do they travel for work, on average? 1. <1 km 2. 1–5 km 3. 5–10 km 4. 10–15 km 5. 15–20 km 6. 20–30 km 7. >30 km Location of off-farm work
B.7	What is the primary food source? 1. Own farm harvest 2. Purchased food 3. Traded food 4. Food gifts 5. Food aid 6. Other (specify)
B.8	What is the secondary food source? (Use codes from question B.7)
B.9	What are the primary crops that you cultivate at home (20% or more of total planted crops)?
B.9	In a good year, how many bags does your household harvest? 1. <5 bags 2. 5–10 bags 3. 10–15 bags 4. 15–20 bags 5. 20–30 bags 6. 30–40 bags 7. >40 bags

**Section C-Financial Capital**	
C.1	Does your household own any of the following? (1 if No, 2 if yes) Radio/Cassette player? Bicycle? Motorcycle/scooter? Refrigerator? Tractor? Plow? Television? Telephone/cell phone?
C.2	What is your average total monthly household income? 1. <E200 2. E200–E500 3. E500–E1000 4. E1000–E2000 5. E2000–E3000 6. E3000–E5000 7. >E5000
C.3	When was the last time someone in your household received money for work (a paycheck), government money, money for services, or payment for product sales? 1. Within the last 3 days 2. Within the last week 3. Within the last 2 weeks 4. Within the last month 5. Within the last 2 months 6. More than 2 months ago

**Section D-Natural Capital**	
D.1	Have you planted anything this season? 1. No 2. Yes
D.2	If no, do you plan to plant this season? 1. No 2. Yes
D.3	If yes, what proportion of your farmland are you currently using for farming? 1. <10% 2. 10%–30% 3. 30%–50% 4. 50%–70% 5. 70%–90% 6. >90%
D.4	What percentage of your farmland did you farm last year? 1. <10% 2. 10%–30% 3. 30%–50% 4. 50%–70% 5. 70%–90% 6. >90%
D.5	Does your household collect wild food? 1. No 2. Yes
D.6	What proportion of your diet comes from wild foods? 1. <10% 2. 10%–30% 3. 30%–50% 4. >50%
D.7	How far do you travel to collect wild foods? 1. <1 km 2. 1–3 km 3. 3–5 km 4. >5 km
D.8	How far do you travel to collect fuel wood? 1. <1 km 2. 1–3 km 3. 3–5 km 4. >5 km
D.9	Please look at the map and outline the following: Where your farmland is located Where you travel to collect firewood Where you travel to collect wild foods

**Section E-Physical Capital**				
E.1	How far (Km) do you travel to reach the [FACILITY]: 1. <1 km 2. 1–5 km 3. 5–10 km 4. 10–20 km 5. 20–30 km 6. 30–50 km 7. >50 km	How do you travel to get to [FACILITY]? 1. Walk 2. Drive 3. Bus/public transport 4. Bicycle	How long does it take you to reach [FACILITY]?	
			Hours	Minutes
Primary School				
Middle School				
Secondary School				
Clinic				
Market				

E.2	How many do you own of each of the following? 1. Chicken 2. Turkey 3. Goat 4. Sheep 5. Cow 6. Donkey 7. Pig 8. Other livestock (specify)
E.3	What is the main source for your drinking water? 1. Tap 2. Well 3. Borehole 4. Running water (river, stream) 5. Lake 6. Other (specify)
E.4	How far is this drinking source from the homestead? 1. Within homestead 2. <0.5 km 3. 0.5–1 km 4. 1–3 km 5. >3 km
E.5	How many months of the year is this water source available? 1. 0–3 months 2. 3–6 months 3. 6–9 months 4. 12 months
E.6	What is the distance to the water sources used for livestock (cattle)? 1. Within homestead 2. <0.5 km 3. 0.5–1 km 4. 1–3 km 5. >3 km

**Section F-Social Capital**	
F.1	Are you or any member of your household a member of a community organization? 1. No 2. Yes
F.2	How many times in the last month did you attend: Church Community Meeting Organizational meeting
F.3	Do you or any household member have any leadership positions in the community? 1. No 2. Yes
F.4	If so, how long have they held the leadership position
F.5	How many times in the last month have you received gifts from friends or community member?
F.6	Does your household trade goods with your neighbors? 1. No 2. Yes
F.7	If, so, how often do you trade, on average? 1. Daily 2. Weekly 3. Every 2–3 weeks 4. Monthly 5. Every 2–3 months 6. 1–2 times per year 7. Other (specify)
F.8	Does your household work for your neighbors? 1. No 2. Yes
F.9	If, so, how often do you work for your neighbors, on average? 1. Daily 2. Weekly 3. Every 2–3 weeks 4. Monthly 5. Every 2–3 months 6. 1–2 times per year 7. Other (specify)
F.10	Do you hire your neighbors? 1. No 2. Yes
F.11	If, so, how often do you hire your neighbors, on average? 1. Daily 2. Weekly 3. Every 2–3 weeks 4. Monthly 5. Every 2–3 months 6. 1–2 times per year 7. Other (specify)
F.12	Where do you get information about farming and weather? 1. Neighbors 2. Community Meetings 3. Government extensions 4. UniSwa 5. Radio/TV 6. Cell phone 7. Newspaper 8. RSSC 9. Aid Organization

**Section G-Health and Food Security**	
G.1	In the past 24 h how many times have household women eaten?
G.2	In the past 24 h how many times have household men eaten?
G.3	In the past 24 h how many times have household children eaten?
G.4	In the past 24 HOURS has anyone in your household eaten the following? (1 if No, 2 if yes) Maize Rice Sorghum Other grains? Bread Chicken Fish Beef Pork Other meat Eggs Beans Vegetables Milk or dairy Other food (specify) │______________________________________________________│
G.5	In the past 7 DAYS has anyone in your household eaten the following? (1 if No, 2 if yes) Maize Rice Sorghum Other grains? Bread Chicken Fish Beef Pork Other meat Eggs Beans Vegetables Milk or dairy Other food (specify) │______________________________________________________│
G.6	How many household children 0–5 years old have died recently (in the past 2 years)?
G.7	(If 0, Skip to next question) By what cause(s)? _____________________________________________
G.8	How many household adults have died recently (in the past 2 years)?
G.9	In the past 30 days, how many household members have visited a hospital or clinic?

**Section H-Drought & Adaptation**	
H.1	How do you know when there is a drought? (What cues, evidence, and trends do you use)? _________________________________________________________________________________________
H.2	Thinking back over the last 30 years (or when you first moved to the community) how have the impacts of drought changed compared to the past? 1. Fewer negative impacts of drought 2. No change in impacts of drought 3. More negative impacts of drought
H.4	In the past 5 years, have you and your household experienced drought? 1. No 2. Yes
H.5	Excluding the last 6 months, has your household done any of the following over the past 5 years in response to drought? (1 if no, 2 if yes) Planting Alternative crops Conservation farming Bee Keeping Raising Indigenous Chickens Collecting natural resources to sell Handicrafts Sending family to work in cities Sending family to work in Sugarcane Participated in training provided by aid organizations Other (specify) ________________________________________________________________________________________
H.6	What were the results of each adaptation (prompt if necessary, increase in production, increased income, increased food consumption, etc.). Planting Alternative crops ________________________________________________________________________________________ Conservation farming ________________________________________________________________________________________ Bee Keeping ________________________________________________________________________________________ Raising Indigenous Chickens ________________________________________________________________________________________ Collecting natural resources to sell ________________________________________________________________________________________ Handicrafts ________________________________________________________________________________________ Sending family to work in cities ________________________________________________________________________________________ Sending family to work in Sugarcane ________________________________________________________________________________________ Participated in training provided by aid organizations ________________________________________________________________________________________ Other (specify) ________________________________________________________________________________________
H.7	In response to drought this year, in the last 6 months, has your household done any of the following? (1 if no, 2 if yes) Planting Alternative crops Conservation farming Bee Keeping Raising Indigenous Chickens Collecting natural resources to sell Handicrafts Sending family to work in cities Sending family to work in Sugarcane Participated in training provided by aid organizations Other (specify) ________________________________________________________________________________________

**Section I-Barriers and Impacts of Adaptation**

For the next section, organize questions based on adaptation behavior. Ask first about adaptations that were done and second about those that weren’t.

If Adaptation Was Done in the Last 6 Months Ask	
I.1	What did [the adaptation] involve, describe what you and your household changed? __________________________________________________________________________________________
I.2	How much money did your household spend to do [the adaptation]? 1. No money spent 3. <E100 4. E100–200 5. E200–500 6. E500–1000 7. >E1000
I.3	Did [the adaptation] require more time spent working or more people? If so, how much _________________________________________________________________________________________
I.4	How has [the adaptation] impacted the following 1. Increased/Improved 2. Decreased/Decline 3. No change Total amount of agricultural production │________________│ Total number of meals eaten by the household │________________│ Amount of food eaten per meal by the household │________________│ Total Household Income │________________│
I.5	Did [the adaptation] have any other impacts to you or your household? ____________________________________________________________________________________
If the Adaptation WASN’T Done, Ask the Following Questions	
I.6	Which of the following BEST explains why you didn’t do [the adaptation]? 1. Don’t know enough about it 2. Don’t have the money 3. Don’t have the time 4. Don’t have the resources (land, plow, household members, etc., specify) 5. It won’t help my household deal with drought 6. No one else is doing the adaptation 7. It is not an option for my household for some other reason (specify) _______________________________________________________________________________________
I.7	Which of the following are additional reasons why you didn’t do [the adaptation]? 1. Don’t know enough about it 2. Don’t have the money 3. Don’t have the time 4. Don’t have the resources (land, plow, household members, etc., specify) 5. It won’t help my household deal with drought 6. No one else is doing the adaptation 7. It is not an option for my household for some other reason (specify) ______________________________________________________________________________________
I.8	Are there other things you would like to be able to do in response to drought that you can’t? Specify________________________________________________________________________________________

## Appendix B. A Qualitative Description of Each Adaptation Included in Our Survey in eSwatini in 2016

B.1 Planting Heat and Drought Resistant Crops

Many households have been attempting this adaptation for many years. The most commonly cited alternative (to maize) crops are sorghum, legumes/beans, cotton (as a cash crop), cassava, and peanut/groundnut. Households report a number of barriers associated with these alternative crops including lack of markets for sale, lack of seeds, lack of sufficient water, lack of knowledge, and lack of labor and land. Some perceived benefits are improved soil quality and decreased pest activity. Most households reported learning about these alternative crops from social networks, aid organizations, or government agents from the Ministry of Agriculture. Very few households reported an increase in income from this adaptation and were unable to provide a clear monetary value from these crops.

B.2 Conservation Farming

Conservation farming and related activities are typically aimed at minimizing soil degradation and erosion. Households report minimize tillage, planting cover crops, and planting legumes as the primary strategies. Most households report learning about these options through aid organizations or the Ministry of Agriculture, however, many households also report a lack of knowledge of proper conservation farming techniques (though many are familiar with the phrase “conservation farming”). Most households who reported successful conservation farming reported an increase in food consumption or agricultural output, however many noted that while this was successful during the past year, it was less successful during the year of the survey.

B.3 Bee Keeping

Very few households reported knowledge of bee keeping as a livelihood strategy. Of the 19 households that reported bee keeping all had learned from a local organization that provided both training and capital to build bee hives/boxes. One household reported success in the previous year; they were able to harvest honey but were unable to sell very much. Most households reported low success due to very low rainfall. Important barriers to bee keeping included lack of knowledge and training, lack of funds/capital to build housing/buy bees, and fear associated with keeping bees near homes and children.

B.4 Chicken Husbandry

There are two strategies for keeping chickens in this region; selling eggs or selling chickens, typically requiring different breeds of chicken. Of the households that reported raising chickens, < 5% reported selling eggs. Those that sold eggs were all part of a community organization with a relationship with restaurants which supported a direct market for egg sales. This organization also provided resources to build chicken coops, feed, and other resources and provided training. At the time of survey, all participants were new the program and had yet to sell any eggs. The majority of household surveys sold chickens directly, rather than eggs. They reported selling opportunistically to neighbors, friends, other community members, and occasionally visitors who lived outside the community. They reported have to buy feed and most housed their chickens in a coop. Primary barriers to adoption were lack of funds to feed chickens or build a coop, concern over a lack of market, and fears regarding chicken theft. Most households reported increased income from this adaptation, having sold a chicken in all cases within the last month and in many cases, the last week. Households reported selling chickens for between E30 and E200 ($3.50–$14).

B.5 Selling Natural Resources

Greater than 90% of households who reported selling natural resources, reported selling firewood. Few households reported selling other natural resources directly but those who did reported selling primarily herbs used for cooking or medicine. Most households reported having to travel between 1 and 10 km (typically walking) to access savanna where they could collect sufficient firewood to sell. While some households reported selling to neighbors and community members, many reported selling on the side of the road to passersby who, most often didn’t reside in the community. As with chicken sales, most households reported selling firewood in the past month and, more often, in the past week for between E15 and E50 ($1–$3.50).

B.6 Selling Handicrafts

Most households that made and sold crafts reported selling items such as mats made from grasses, traditional clothing and decorations, wood products and occasionally, kitchen items, jewelry and decorative bowls. Households reported being self-taught and getting training from local organizations for craft making and small business management. In some cases households also reported receiving start-up funding from local organizations. Households report a lack of start-up capital or lack of skills as the primary barriers. Because of the wide range of potential handicraft items sold, households reported a wide range of income from sales (ranging from E30 to E500) depending on the item. Handicraft sales tend to be less consistent than chicken or firewood sales, most households reported making a sale in the past year.

B.7 Off-Farm Work

Many households reported attempting to find salaried employment off farm, primarily in the agricultural industry, at local schools, in the nearby cities (in shops) and in cities in South Africa. Many report a lack of employment opportunities and a lack of labor in the household. For those that are successful, incomes vary significantly (E250/month–E20,000/month, $17–$1400).

B.8 Participation in Local Organizations

Participation in local organizations was the second least common adaptation. Organizations that households participated in included those run by NGO’s aimed at a specific type of training or capacity building, local savings and loan organizations (where participants pay money each month and larger sums are disbursed over time depending on need), and larger local organizations aimed at a project or goal (such as raising funds for a school or well). Participation often requires monthly dues (usually less than E20) and monthly meeting participation. The primary barrier to participation is a lack of availability (some NGOs don’t work in all communities) or some other restriction (e.g., only for people of a certain age, women). Households primarily report gaining access to training, funds for other adaptations, and the potential to borrow physical capital through these organizations.

## Appendix C

**Table A1:** List of the most commonly cited barriers of adaptation for each adaptation studied.

Adaptation	Most Commonly Cited Barrier
Planting drought/heat resistant crops	Insufficient money/income
Conservation farming	Insufficient training/knowledge
Bee keeping	Insufficient training/knowledge
Chicken husbandry	Insufficient money/income
Selling natural resources	Insufficient Market/Infrastructure
Selling crafts	Insufficient training/knowledge
Other salaried off-farm work	Insufficient household labor available
Participating in local organizations	Insufficient opportunities/availability
