# Supplementary figures and images for: Climate-Driven Adaptation, Household Capital, and Nutritional Outcomes among Farmers in Eswatini
Source: Int J Environ Res Public Health. 2019 Oct 23;16(21):4063. doi: 10.3390/ijerph16214063 (PMC6862074; doi:10.3390/ijerph16214063)

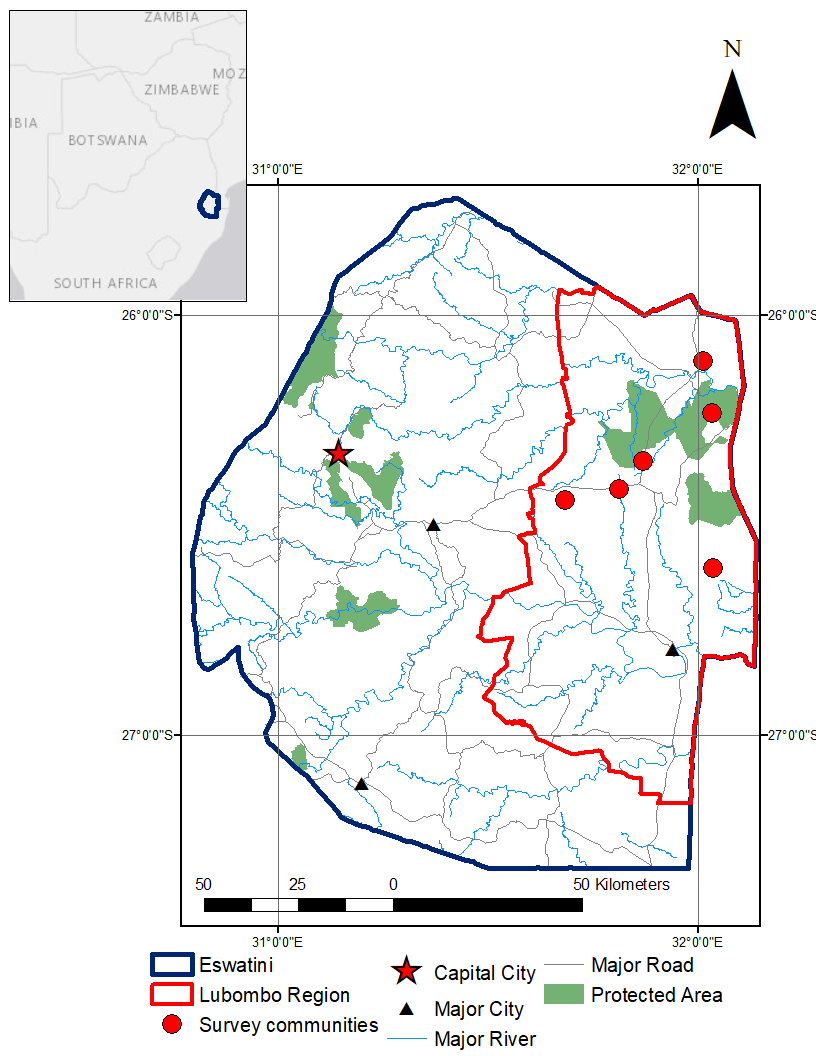

Supplement: Supplementary File 1 [file ijerph-16-04063-s001.zip › Figure 1.png]

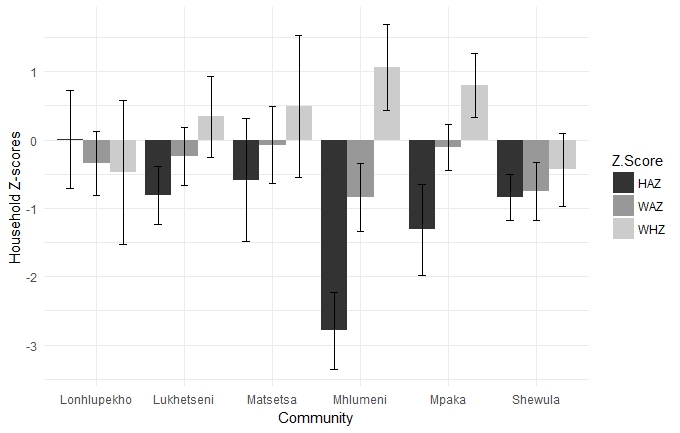

Supplement: Supplementary File 1 [file ijerph-16-04063-s001.zip › FIgure 2.jpeg]

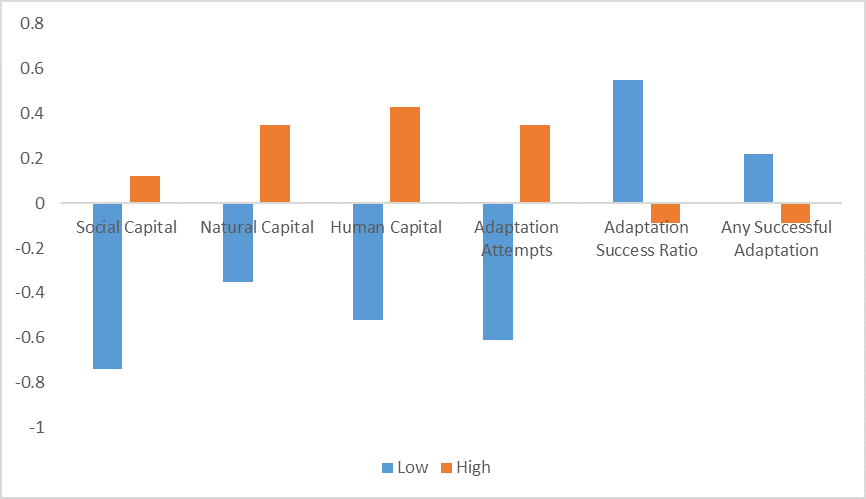

Supplement: Supplementary File 1 [file ijerph-16-04063-s001.zip › Figure 3.png]

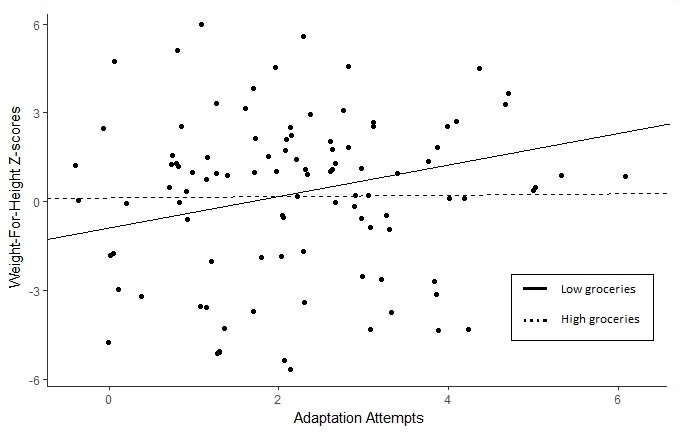

Supplement: Supplementary File 1 [file ijerph-16-04063-s001.zip › Figure 4.jpg]

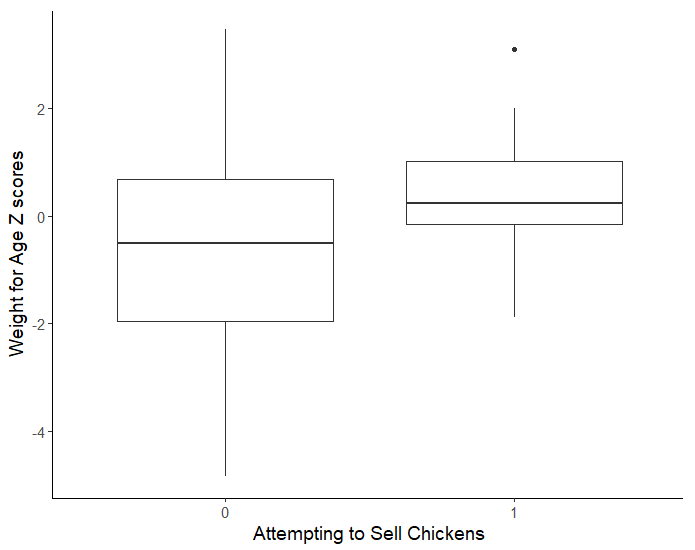

Supplement: Supplementary File 1 [file ijerph-16-04063-s001.zip › Figure 5.png]

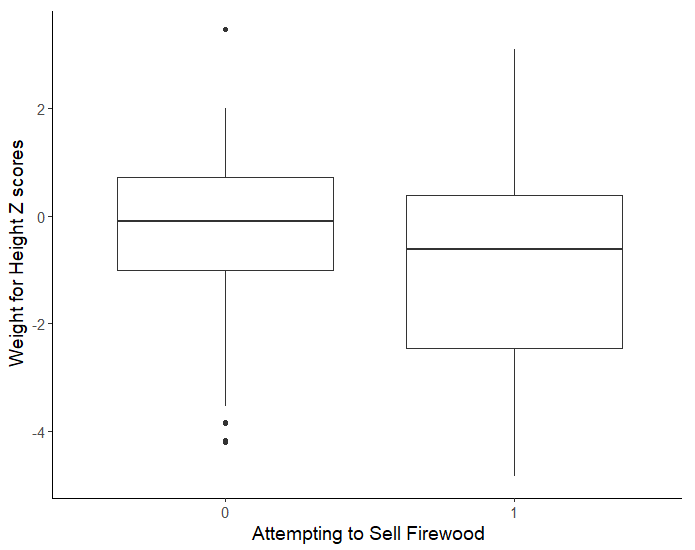

Supplement: Supplementary File 1 [file ijerph-16-04063-s001.zip › Figure 6.png]
